# Arthroscopic management of rockwood type V acromioclavicular joint dislocation using a modified suture-passage technique with a four-leaf clover plate and adjustable loop plate: a case report

**DOI:** 10.3389/fsurg.2025.1616395

**Published:** 2025-11-10

**Authors:** Dejie Zhou, Xingyong Qi, Xinwei Liu, Yaqin Li, Bo Liu, Yongwen Zhou, Hantao Liu, Fan Zhang, Lianghu Zhao, Cai Liu

**Affiliations:** 1 Department of Orthopedics, The Affiliated Hospital of Panzhihua University, Panzhihua, China; 2 Department of Orthopedics, Huidong County Traditional Chinese Medicine Hospital, Liangshan Yi Autonomous Prefecture, Sichuan, China; 3 Department of Physical Examination Center, The Affiliated Hospital of Panzhihua University, Panzhihua, China

**Keywords:** acromioclavicular joint, joint dislocation, modified suture-passage technique, four-leaf clover plate, adjustable loop plate, arthroscopy

## Abstract

**Introduction:**

Acromioclavicular (AC) joint dislocation is a prevalent shoulder injury that, if not treated promptly and appropriately, can cause persistent pain and functional impairment. Numerous surgical techniques have been described, but no universally accepted gold standard exists. Arthroscopic coracoclavicular fixation with a TightRope has gained popularity owing to its minimally invasive approach, enhanced visualization, reliable mechanical stability, and ability to replicate coracoclavicular ligament biomechanics. However, potential complications such as coracoid or clavicular fractures remain concerns.

**Case presentation:**

A 63-year-old male was admitted 12 days after a fall, presenting with left shoulder pain and limited mobility. Physical examination revealed marked superior displacement of the distal clavicle, a positive piano-key sign, and no significant anteroposterior translation. Radiographs confirmed a Rockwood type V AC joint dislocation. Arthroscopic coracoclavicular fixation was performed using a modified suture-passage technique with a four-leaf clover plate and an adjustable loop plate (ALP). At three months, the patient had resumed normal activities. Though clavicular tunnel enlargement was detected at 3 months and showed slight progressed by 6 months, it subsequently stabilized thereafter without any consequence at one-year follow-up. Only a minor loss of acromioclavicular joint reduction was observed, and the patient remained asymptomatic with good functional recovery.

**Conclusions:**

This modified arthroscopic technique using a four-leaf clover plate with an ALP showed favorable short-term outcomes in AC joint dislocation. Further studies are required to confirm its long-term safety and effectiveness.

## Introduction

Acromioclavicular (AC) joint dislocation accounts for 9%–12% of all shoulder injuries, particularly among individuals involved in traffic accidents or those with high levels of physical activity (1–3). To date, more than 150 surgical techniques have been described for its management (4). Among them, arthroscopic coracoclavicular ligament repair using loop or suspensory devices has gained increasingly popularity due to its minimally invasive nature, reliable biomechanical properties, low complication rates, and the advantage of avoiding secondary implant removal (3, 5–12).

Nevertheless, complications such as clavicle and coracoid fractures remain a major concern, often leading to fixation failure (6, 8, 13–18). These adverse events are closely related to bone tunnel design, particularly tunnel diameter, number, orientation, and positioning (3, 16, 19, 20). In the traditional TightRope system (Arthrex), the oblong coracoid button is advanced through clavicle and coracoid tunnels using a Nitinol Suture Passing Wire (Arthrex), flipped into position under arthroscopic guidance, and secured with a round clavicle button on the superior clavicle (6). To allow passage of the oblong button (3.5 mm in diameter), a tunnel diameter of at least 4.0 mm is required (3, 6, 7, 21). However, the need for such large tunnels, combined with the potential for misalignment during drilling, may compromise bone integrity and increase the risk of iatrogenic fractures, with reported fracture rate of up to 6% (3, 16, 17).

The study aims to introduce a minimally invasive, arthroscopically guided fixation technique using a four-leaf clover plate combined with an adjustable loop plate, designed to reduce tunnel-related complications while maintaining stable fixation in the treatment of AC joint dislocation.

## Case presentation

A 63-year-old male farmer presented with left shoulder pain and limited mobility12 days after a fall. He took loxoprofen sodium (60 mg, three times daily for three days) without relief and reported no relevant medical, family, or psychosocial history.

On examination, there was marked contusion and bruising around the shoulder without skin laceration. Local tenderness was noted over the AC joint, with the distal clavicle visibly displaced superiorly. The piano-key sign was positive, and reduction was achievable with manual pressure. The Constant-Murley score was 13 (22). Radiographs (anteroposterior and tangential clavicle views) revealed a Rockwood type V AC joint dislocation with >100% superior displacement of the distal clavicle. Other laboratory and preoperative evaluations were unremarkable (23).

Surgery was performed under General anesthesia by an experienced orthopedic surgeon (Dr. Zhou Dejie). Prophylaxis included intravenous cefazolin (1 g) and tranexamic acid (2 g). The patient was positioned in the lateral decubitus position, and a 30-degree arthroscope was introduced via the posterior portal for diagnostic arthroscopy. Anterolateral portal was established to clear the rotator cuff interval and partially expose the base of the coracoid process. The arthroscope was then shifted through the posterior portal into the subacromial space, a lateral portal was created for further cleaning of the subacromial space. The arthroscope was introduced through the lateral portal into the subacromial space, and an anterolateral portal was established to clear the anterior rotator cuff interval, fully exposing the base of the coracoid process. Arthroscopically, the dislocated AC joint was visible.

**Figure 1:**
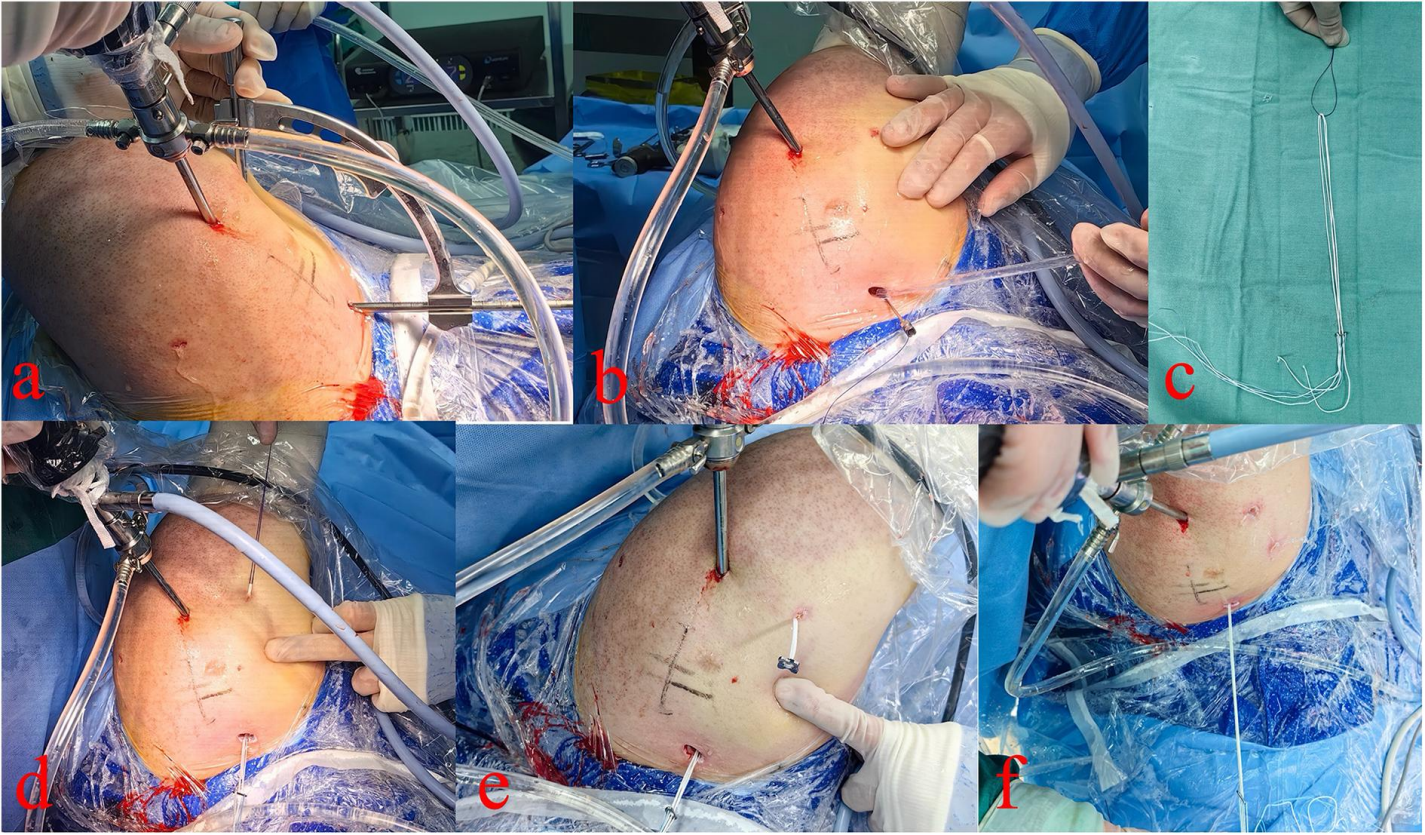
Arthroscopic extra-articular view. **(a)** Locator used for clavicular and coracoid bone tunnel creation. **(b–d)** Traction sutures introduced and loop advanced under arthroscopic guidance. **(e,f)** Four-leaf clover plate secured on the loop and positioned beneath the coracoid process.

**Figure 2:**
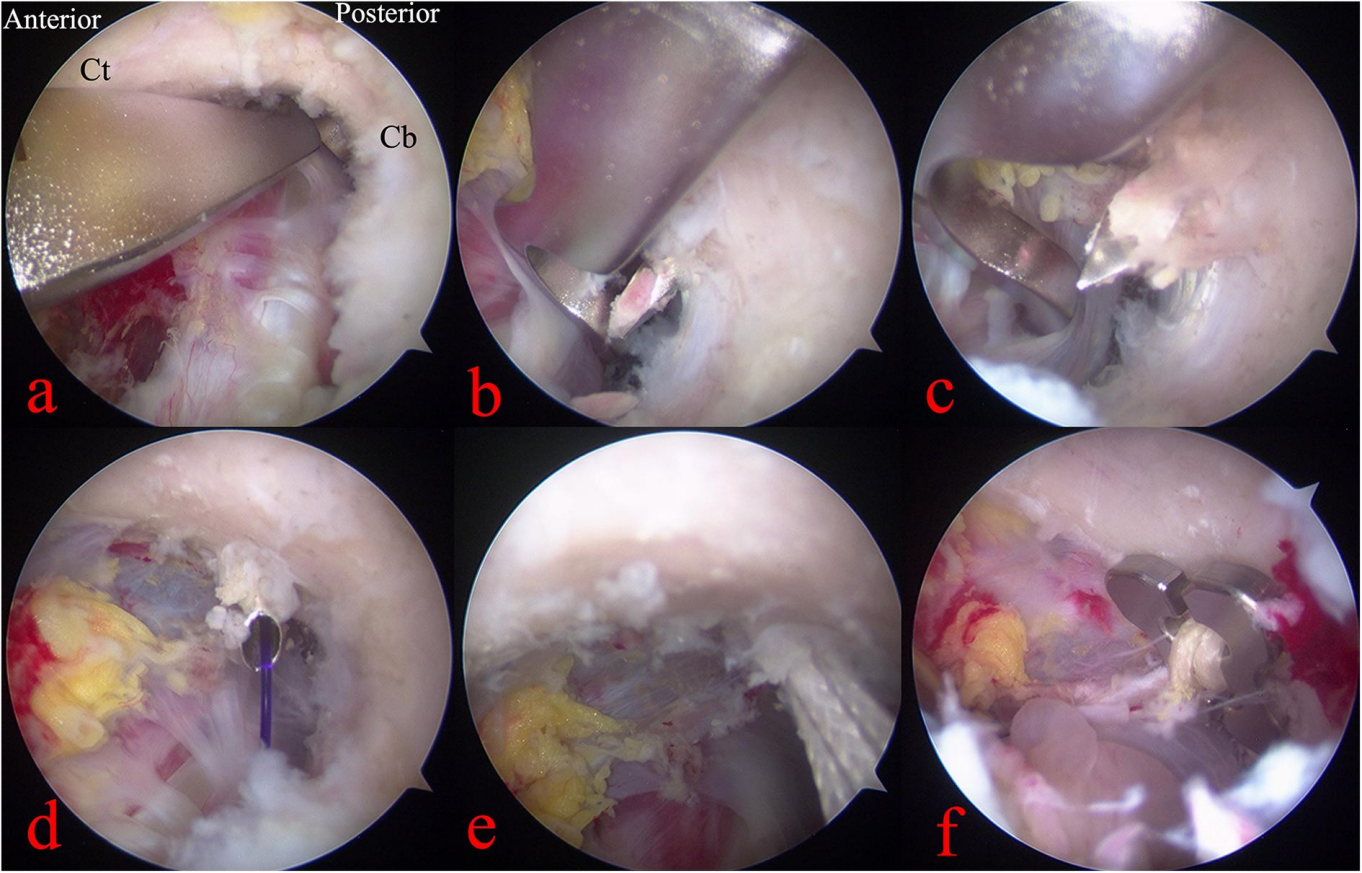
Arthroscopic intra-articular view (Ct, coracoid tip; Cb, coracoid base). **(a–c)** Locator positioning and tunnel preparation with Kirschner wires. **(d,e)** Traction sutures introduced and loop advanced. **(f)** Four-leaf clover plate positioned beneath the coracoid base.

A 1 cm incision was made approximately 3 cm (about 25% of the clavicular length from the clavicle's distal end) from the distal clavicle to expose the superior surface (24). Under arthroscopic guidance, a targeting device was inserted at the midpoint of the medial and lateral sides of the coracoid base, and the external portion of the targeting device was positioned at the midpoint of the anterior and posterior aspects of the superior clavicular surface under direct visualization (Figures 1, 2). After the reduction of the AC joint by an assistant, and the bone tunnel was located at the central position of the coracoid base, a 2.0-mm Kirschner wire was used from the superior surface of the clavicle toward the coracoid base. Then, the tunnel was enlarged using a 2.5-mm Kirschner wire. A polydioxanone suture (PDS) was introduced into the joint via spinal needle, and retrieved through the anterolateral portal, and used to shuttle a second traction PDS loop attached to the adjustable loop plate (ALP, Chunli, Beijing, China). The loop was retrieved outside the body, where the four-leaf clover plate (Chunli, Beijing, China) was threaded and secured (Figure 1). Under arthroscopic guidance, the construct was drawn back so that the clover plate engaged the inferior coracoid surface while the ALP rested on the superior clavicle.

**Figure 3:**
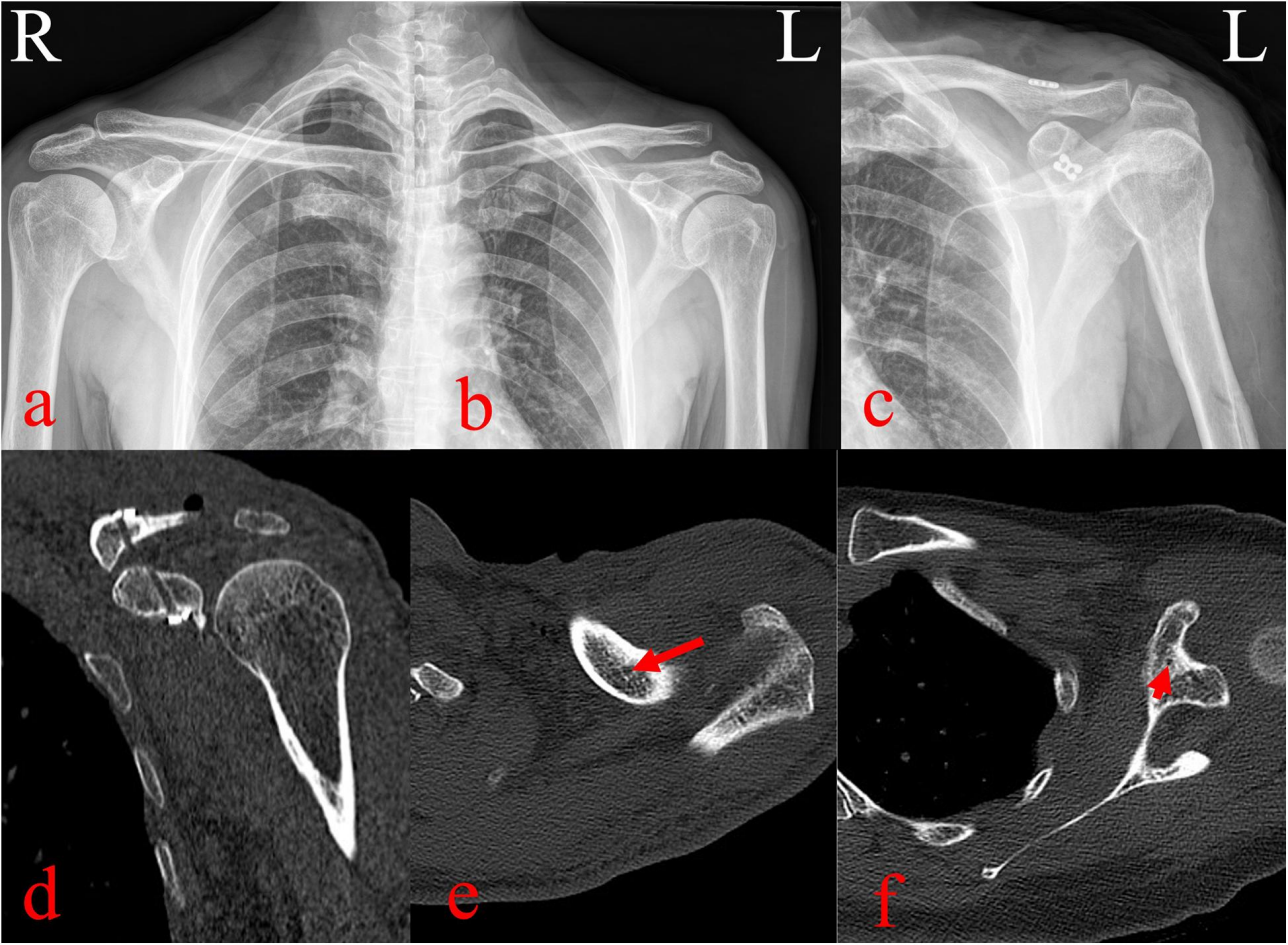
Pre- and postoperative imaging evaluation. **(a–b)** Preoperative radiographs showing >100% displacement of the left AC joint. **(c)** Postoperative radiographs showing anatomic reduction with appropriate placement of the ALP and four-leaf clover plate. **(d–f)** Three-dimensional computed tomography reconstructions confirming central positioning of clavicular and coracoid bone tunnels with satisfactory implant placement.

The AC joint was manually reduced, and the loop was tensioned and tied. C-arm fluoroscopy confirmed complete reduction of the AC joint. The x-rays and three-dimensional computed tomography was taken the day after surgery (Figure 3). The incisions were disinfected and closed in layers. Postoperatively, the oral loxoprofen (60 mg) were administered three times daily for three days. Rehabilitation included passive motion from day one, brace immobilization for four weeks, initiation of active exercises at six weeks, and return to normal activities at three months. Follow-up visits were conducted with x-rays at 3, 6 and 12 months postoperatively, during which the shoulder joint functional recovery and visual analog scale (VAS) were meticulously documented.

**Figure 4:**
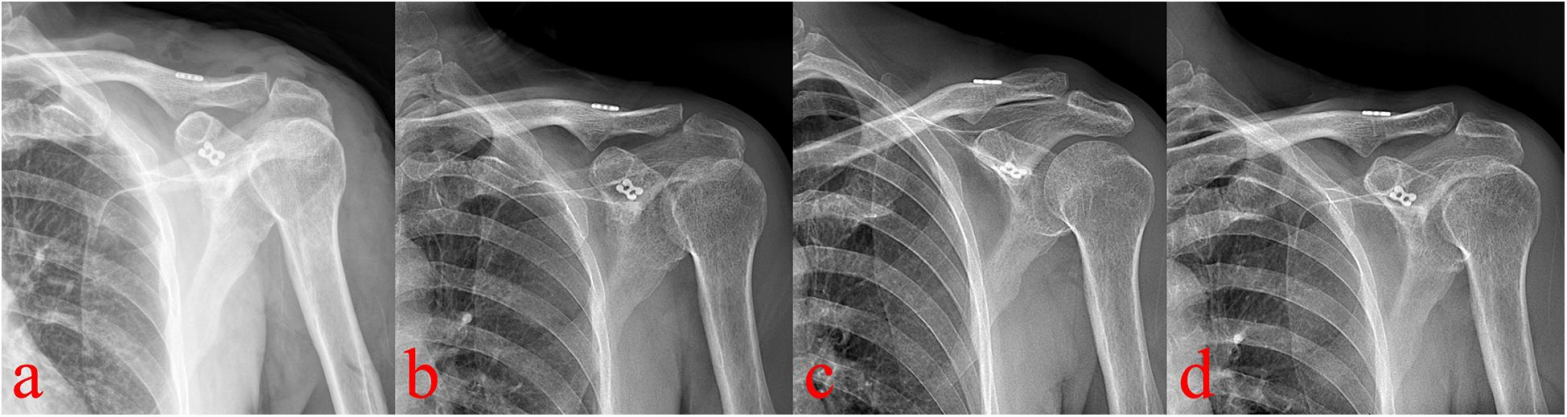
Postoperative radiographs (**a–d**, immediate postoperative, 3-month, 6-month, and 1-year) show progressive clavicular tunnel enlargement until 6 months, then stabilization, with no coracoid tunnel widening. A minor loss of AC joint reduction observed.

Pain decreased from a VAS score of 6 preoperatively to 2 on day three, and to 0 at three months. At one year, the Constant-Murley score was 98. Tunnel enlargement was observed at 3 months, progressed slightly by 6 months, and stabilized thereafter without clinical consequence. At the 1-year follow-up, the clavicle tunnel showed no further widening compared with the 6-month findings. A minor loss of acromioclavicular joint reduction was noted, and the patient remained asymptomatic with good functional recovery (Figure 4). The Constant-Murley score reached 98, and serial radiographs confirmed reduction of the AC joint without redislocation or complications such as clavicular or coracoid fractures.

## Discussion

Currently, suspension fixation has become a widely accepted method for the treatment of AC joint dislocation. Reported suspension techniques include suture fixation, GraftRope, synthetic artificial ligament, EndoButton with suture, TightRope, Weaver-Dunn coracoacromial ligament transfer, and autologous tendon grafts (3, 25). To the best of our knowledge, no previous study has reported arthroscopic coracoclavicular fixation using a four-leaf clover plate combined with an ALP through bone tunnels of such small diameter. In this report, fixation was achieved arthroscopically using a modified suture-passing technique, gradual tunnel enlargement, and reduced bone tunnel diameter.

By minimizing the tunnel diameter and modifying the suture-passing technique, the procedure eliminates the need to flip the loop plate, thereby reducing the risk of iatrogenic clavicle and coracoid fractures during both early and late postoperative periods. Traditional loop plate requires a tunnel diameter of at least 4.0 mm to accommodate the passage and flipping the of the plate, with reported fracture rates of up to 6% at the clavicle and coracoid process (3, 6, 7, 21). In contrast, the present technique requires only a 2.5 mm tunnel for passage of the adjustable loop, thereby reducing the risk of secondary fractures. This is consistent with previous findings that smaller drill holes result in less compromise of bone integrity (26–29).

Tunnel widening remains a major concern and is closely related to fractures in coracoclavicular reconstruction. Previous study has reported that, regardless of whether the bone tunnel was positioned medially or laterally, nearly all patients exhibited varying degrees of tunnel enlargement at the 6-month follow-up (30). Another study demonstrated no-healing and progressive enlargement of bone tunnels, with up to 82% of cases, with an averaging 2 mm (range, 1–4 mm) at a mean follow-up of 281 days (17). Tunnel enlargement has been recognized as a risk factor for delayed fractures of the clavicle and coracoid (8, 18, 24, 28, 31). In the present case, the bone tunnel was created suing a 2.0-mm Kirschner wire for initial positioning, followed by enlargement with a 2.5-mm wire. Theoretically, even if subsequent widening reached the average 2 mm reported in the literature, the final diameter would be approximate 4.0 mm—the minimum required for the traditional loop plate passage. Our follow-up confirmed this inference: the clavicular tunnel enlarged from 2.5 mm postoperatively to 3.6 mm at 3 months, and 4.2 mm at 6 months, after which no further significant changes were observed. Similarly, the coracoid tunnel remained stable without marked widening at 1 year.

Although more than 150 fixation techniques for AC joint have been described, no consensus gold standard exists (4). Suspension fixation methods most commonly utilize single- or double-tunnel constructs. Biomechanical studies have suggested that double-tunnel reconstruction provides greater resistance to anterior, posterior, and superior displacement of the clavicle, more closely reproducing the native coracoclavicular ligament function (32). However, other investigations have demonstrated no significant biomechanical differences among single-tunnel adjustable loop constructs, double-plate fixation, and triple-button techniques under translational loading (5–70 N) and load-to-failure testing, with all outperforming controls (12). Moreover, under 70 N of multidirectional loading, single-tunnel fixation was shown to achieve biomechanical properties comparable to those of double-tunnel constructs, while theoretically reducing the risk of tunnel-related clavicular fracture (33). In addition, the single-tunnel technique offers the advantages of less cost and technical simplicity making it easier to learn and perform.

## Conclusion

In conclusion, we report a single case of arthroscopic-assisted coracoclavicular fixation for AC joint dislocation using a targeting device, reduced tunnel diameter, modified suture-passing technique, and a four-leaf clover plate combined with an ALP. While this technique appears to provide a minimally invasive and potentially safer alternative with fewer complications, the evidence remains preliminary. Given the inherent limitations of a single-case report and the relatively short follow-up period, these findings should be interpreted with caution. Further studies involving larger patient cohorts and extended follow-up are essential to validate the safety, reproducibility, and long-term efficacy of this modified approach.

## Data Availability

The raw data supporting the conclusions of this article will be made available by the authors, without undue reservation.
